# Pathway to mental health recovery: a qualitative and quantitative study on the needs of Chinese psychiatric inpatients

**DOI:** 10.1186/s12888-016-0959-6

**Published:** 2016-07-12

**Authors:** B. W. M. Siu, M. M. Y. Tsang, V. C. K. Lee, A. C. Y. Liu, S. Tse, H. S. M. Luk, N. K. Y. Lo, P. H. Lo, Y. L. Leung

**Affiliations:** Department of Psychiatry, Castle Peak Hospital, Hong Kong, SAR People’s Republic of China; The Mental Health Association of Hong Kong, Hong Kong, SAR People’s Republic of China; Department of Social Work and Social Administration, Associate Dean at Faculty of Social Sciences, The University of Hong Kong, Hong Kong, SAR People’s Republic of China; Department of Forensic Psychiatry, Castle Peak Hospital, 15 Tsing Chung Koon Road, Tuen Mun, New Territories Hong Kong

**Keywords:** Information, Participation, Mental Health Recovery, Inpatients, Chinese

## Abstract

**Background:**

Exploration of the information and participation needs of psychiatric inpatients is an important step for the implementation of recovery-oriented mental health service. The objective of this study was to explore the information and participation needs of Chinese psychiatric inpatients in the largest psychiatric hospital in Hong Kong.

**Methods:**

The study was divided into two parts. In the first part, eight focus groups with patients, patients’ relatives and healthcare professionals were held to identify 22 items of information needs and 16 items of participation needs of Chinese psychiatric inpatients. Basing on the items identified in the first part of the study, a questionnaire was developed to survey on the importance of the different information and participation needs in the second part of the study. Participants were asked to rate in rank order their perceived importance of the items in the questionnaire survey.

**Results:**

A hundred and eighty three Chinese psychiatric inpatients completed the questionnaire and the majority of them suffered from schizophrenia (68.3 %). For information needs, the top three needs rated by patients as the most important in descending order were: “Information on the classifications of mental illnesses, signs and symptoms and factors contributing to relapse”, “Information on the criteria and arrangements for discharge”, and “Information on the importance of psychiatric drug taking and its side effects”. For participation needs, the top three needs rated by patients as the most important in descending order were: “Enquiring about personal needs and arrangements”, “Keeping in touch with the outside world”, and “Learning and practising self-management”.

**Conclusions:**

This study reveals that Chinese psychiatric inpatients are concerned about information on their mental illness and its treatments as well as the criteria for discharge. On the other hand, patients are concerned about their personal needs, their self-management, as well as their keeping in touch with the outside world during their hospitalisation. Moreover, patients with different socio-demographic and clinical characteristics have different information and participation needs. The results of the present study serve as a reference for designing guidelines, strategies, and programmes to meet the information needs and participation needs of psychiatric inpatients in Hong Kong.

**Electronic supplementary material:**

The online version of this article (doi:10.1186/s12888-016-0959-6) contains supplementary material, which is available to authorized users.

## Background

### Mental health recovery

Mental health recovery is a journey of healing and transformation enabling a person with a mental disability to live a meaningful life in the community of his or her choice, while striving to achieve his or her full potential [1]. As an essential direction of mental health care reform and a move towards continuous service quality improvement, the concept of recovery-oriented care has already been incorporated into the national policies, service standards and guiding principles for the transformation of the mental health systems in many countries, including the United Kingdom, New Zealand, Australia, America, and Canada [2–7].

### Recovery-oriented services

Recent systematic review strongly supports the view that hospital-based facilities for individuals with severe mental illnesses should deliver “recovery-oriented” services [8]. Moreover, there is evidence supporting the idea that implementation of recovery-oriented services could increase the satisfaction of patients towards the services delivered to them [9, 10]. William Anthony has proposed a widely quoted definition of recovery as ‘a deeply personal, unique process of changing one’s attitudes, values, feelings, goals, skills and roles. Recovery involves the development of a new meaning and purpose in one’s life as one grows beyond the catastrophic effects of mental illness’ ([11], p.21). This definition of recovery emphasised that recovery is an individualised process and that patients should be empowered, and their strengths should be explored in order for them to gain a new valued role in life despite the detrimental effects of mental illness. Also, as recovery is a personal and individualised process, patient’s participation in the treatment and management plan is important for the development of a treatment plan tailor-made for the patient. Farkas and colleagues concurred with the definition of recovery by William Anthony that recovery-oriented evidence-based mental health programmes should be grounded on the notion that a majority of people can grow beyond the catastrophe of a severe mental illness and lead a meaningful life in their own community [12]. Among the different strategies in the implementation of recovery-oriented services, the promotion of “patient empowerment” and “patient’s participation” are perceived to be of upmost importance [1, 13–15].

### Information and participation needs

“Patient empowerment” means “the provision of information regarding therapeutic options so that a patient can actively participate in the decision on whether to undergo a diagnostic or therapeutic procedure, or pursue other alternatives” [16, 17]. In a focus group study of patients in acute psychiatric wards conducted in the United Kingdom, it was concluded that information is the key to patient empowerment and fulfilling the information needs of psychiatric inpatient is essential for the development of patient-centred care [18]. On the other hand, high quality patient-centred care should address the information needs of psychiatric inpatients. A survey conducted in Sweden on two different samples of psychiatric inpatients revealed that effort should be put on the area of patient information as one of the targets for the development of quality assurance programmes [19]. For “patient’s participation”, the involvement of patients in organisational service planning and clinical governance processes has been highlighted [8]. In addition to collaboration in their care, patients’ participation in developing, running and reviewing services has been shown to have positive outcomes for both the services and the patients who take on these roles [9, 10]. Addressing the participation needs of psychiatric inpatient has been a major focus for the delivery of quality mental health services in the recent years [20].

### Traditional view in Chinese setting

The traditional healthcare service delivery in the Chinese populations emphasises “paternalism” or “maternalism” and healthcare professionals tend to provide information to psychiatric inpatients basing on their own perspective and prescribe the management plans for patients with the patients being relatively passive and less involved in decisions about his or her own treatment and rehabilitation process [21, 22].

### Recovery-oriented services in Asians

Despite a long history of recovery-oriented services being implemented in mental health settings in a number of countries, recovery-oriented practice is relatively novel in the mental health systems serving Chinese populations. In Asian countries such as mainland China and Taiwan, recent service reform towards community mental health care and the focus on the strengths and resources of patients as well as patients’ self-help are in line with the delivery of recovery-oriented mental health service [22–26]. In Hong Kong, an Asian city, it is only in recent years that recovery-oriented practice has been promoted as one of the directions of mental health service reform. To demonstrate this progress, peer specialists, for example, are employed in different mental health units in Hong Kong [21, 27–29]. Because of the traditional view on healthcare service delivery as mentioned above, despite this progress of employing peer specialists, meeting the information needs and participation needs of psychiatric inpatient is still a challenge for the Chinese populations. The investigation of the information and participation needs of Chinese psychiatric inpatients shall be one of the initial steps in fulfilling their needs as well as for the implementation of recovery-oriented mental health services with Chinese populations.

### Objective of this study

As the concept of recovery is relatively new in the Chinese populations and different researchers have different definitions on recovery, it is worthwhile that this concept in the context of information and participation needs be explored in both qualitative and quantitative ways [1–7, 11, 12].

This study set out to explore the information and participation needs of Chinese psychiatric inpatients by focus group discussion and questionnaire survey in Castle Peak Hospital, the largest psychiatric hospital in Hong Kong. Prior to this study, there had been no local study focusing on the exploration of these areas for psychiatric inpatient.

## Methods

This is a qualitative and quantitative study on the needs of Chinese psychiatric inpatients with focus on their information and participation needs. This study was divided into two parts. The first part (the qualitative part) was to identify a pool of items of information and participation needs of Chinese psychiatric inpatients by focus group discussion involving psychiatric inpatients, patients’ relatives and healthcare professionals. A survey questionnaire was then developed basing on the pool of items identified in the focus group discussions. In the second part (the quantitative part) of the study, a group of Chinese psychiatric inpatients were recruited as participants. They were asked to rate in rank order their perceived importance of the items in the survey questionnaire.

### Ethics, consent and permissions

In Hong Kong, Castle Peak Hospital provides inpatient, out-patient, and community psychiatric services for people residing in Tuen Mun area and Yuen Long area serving a population of more than a million. Tuen Mun and Yuen Long constituted the New Territories West Cluster, according to the division of the Hospital Authority of Hong Kong. Approval to conduct the study was granted by the Ethics and Research Committee of the New Territories West Cluster of the Hong Kong Hospital Authority. All the focus group members in the first part of the study and all the participants of the second part of the study had signed a written informed consent for their participation in this study.

### Focus groups

Eight focus groups were held with 46 members to collect their views on information needs and participation needs of Chinese psychiatric inpatients. Members were inpatients of the acute wards (*n* = 8), medium-stay wards (*n* = 15), and rehabilitation wards (*n* = 10); patients’ relatives (*n* = 6); and healthcare professionals (*n* = 7) of Castle Peak Hospital. Table 1 shows the socio-demographic characteristics of the focus group members.

**Table 1:** Demographic characteristics of focus group participants

	Number	Percentage
Professional group total	7	
Gender		
Male	3	42.86
Female	4	57.14
Professional group		
Medical	1	14.29
Nursing	3	42.86
Occupational therapy	1	14.29
Medical social work	2	28.57
Years of experience in psychiatric services		
0–10 years	1	14.29
11–20 years	3	42.86
> 20 years	3	42.86
Patient group total	33	
Gender		
Male	21	64.10
Female	12	35.90
Age		
18–30	3	7.69
31–40	9	23.08
41–50	6	20.51
51–60	13	35.90
> 60	2	12.82
Primary diagnosis		
Schizophrenia	28	87.18
Depression/Bipolar affective disorder/Schizoaffective disorder	5	12.82
Education Level		
No formal education	1	5.13
Primary	3	12.82
Secondary	26	74.36
Tertiary	3	7.69
Current wards/units		
Acute wards	8	23.08
Medium-stay wards	15	41.03
Rehabilitation wards	10	35.90
Relative group total	6	
Gender		
Male	2	33.33
Female	4	66.67
Age		
18–40	0	0.00
41–60	4	66.66
> 60	2	33.33
Education Level		
No formal education	0	0.00
Primary	2	33.33
Secondary	2	33.33
Tertiary	2	33.33
Relationship with the patient		
Patient’s parent	4	66.67
Patient’s child	1	16.67
Patient’s sibling	1	16.67

In the focus group, group members discussed on the importance of information provision (e.g., patient charter, patient’s rights and responsibilities, ward facilities, mental health educational materials related to mental illness and effects and side effects of medications, discharge information, hotlines services, and self-help groups) and patient’s participation (e.g., in treatment plan, in the development of ground rules of wards, and in the improvement of the ward environment and services) for Chinese psychiatric inpatients. In the focus group discussion, the investigators acted as moderators and research assistants as co-moderators to guide the discussion according to a list of guiding questions and a list of non-directive probes as shown in the focus group discussion guide (Additional file 1).

In the focus group discussion, linguistic features were not the main focus. The discussion in fact served as a “verbal questionnaire” for exploring the group members’ responses prompted by the guiding questions in the focus group discussion guide. Group members’ responses were audio-taped and then transcribed by the investigator (MMYT, VCKL, HSML) of each group for analysis. In order to ensure the accuracy of the transcription, another investigator (NKYL, PHL, YLL) was responsible for checking the transcripts by reviewing the audio-tapes of each group.

The first step of analysis was to identify different items reflected by the transcripts concerning information and participation needs. An item could be a phrase, a sentence or a paragraph embodying ideas about information and participation needs. Starting from an inductive approach, the investigators (MMYT, VCKL, HSML, NKYL, PHL, YLL) read through the transcripts to identify the items that were relevant to information and participation needs and consensus was made in a face-to face group discussion among the investigators (MMYT, VCKL, HSML, NKYL, PHL, YLL). There had been no explicit criteria for rating an item as relevant and the investigators determined that an item was relevant in the face-to-face group discussion basing on their clinical experience. All the investigators had more than 5 years of clinical experience in psychiatric inpatient setting.

The second step followed a deductive approach by grouping the identified items to form categories with reference to the available literature. An initial categorisation system was then formulated by an investigator (VCKL). Transcripts of each of the focus groups were reviewed again by the investigator (MMYT, VCKL, HSML) of each group and another investigator (NKYL, PHL, YLL) to ensure the relevance and representativeness of the categorisation system for the content of the transcripts. A pool of items for information provision and patient’s participation were identified. For each of the identified item, all the investigators agreed that the item was relevant/highly relevant and are representative/highly representative in describing either the information needs or participation needs of Chinese psychiatric inpatients.

### Development of survey questionnaire

A total of 22 items of information needs and 16 items of participation needs of Chinese psychiatric inpatients were identified from the focus group discussion (Table 2).

**Table 2:** Information needs and participation needs derived from focus group discussion

Information needs (22 items)	Participation needs (16 items)
Information on the classifications of mental illnesses, signs and symptoms and factors contributing to relapse	Collaborating with professionals in setting treatment and care plans
Information on the reasons for in-patient admission and related ordinance	Learning and practising self-management
Information on different residential placements	Enquiring about personal needs and arrangements
Information on daytime training placements after discharge from in-patient setting	Providing opinion on how to improve the services and facilities of ward
Information on the criteria and arrangements for discharge	Keeping in touch with the outside world
Information on the ways to raise self-image and overcome stigma adhered to people with mental illnesses	Expressing needs and following up whether needs are meet
Information on hospital fees and application of financial assistance	Understanding the treatment and rehabilitation plans and providing enough information for healthcare professionals to take reference
Information on the reasons behind the hospital rules and regulations	Learning from other patients
Information on the daily routine and schedules of ward	Reporting discomfort actively
Information on how to maintain good physical health	Expressing personal feelings
Information on the arrangements at different stages of rehabilitation including pre-discharge arrangements	Preparing for the interview with healthcare professionals
Information on the news from the outside world	Learning how to cope with stress and prevent relapse
Information on patient’s right (including ways of expressing opinion and making enquiries)	Participating in vocational training
Information on how relatives can accept, participate and help	Participating in social activities
Information on job acquisition	Participating in training of domestic and self-care skill
Information on stress management and relapse prevention	Learning how to take care of others
Information on alcohol and substance misuse	
Information on the schedule of meetings with healthcare professionals	
Information on community services and resources	
Information on the different kinds of care and rehabilitation services	
Information on medication prescriptions	
Information on the importance of psychiatric drug taking and its side effects	

Basing on the item pool obtained on information provision and patient’s participation in the focus group discussion, a survey questionnaire was developed (Additional file 2).

### Validation of the survey questionnaire

The survey questionnaire was developed basing on the focus group discussion among Chinese psychiatric inpatients, patients’ relatives and healthcare professionals and its face validity with cultural adaptation was established. The content validity of the questionnaire was established by the agreements among investigators who were healthcare professionals on its relevance and representativeness in assessing the information and participation needs of Chinese psychiatric inpatients. Factor analysis was not performed for further testing the construct validity of the questionnaire and the test -retest reliability as well as the sensitivity to changes of the questionnaire were not tested in this study. The validity and reliability of the survey questionnaire are yet to be further explored in future studies with larger samples.

### Participants of questionnaire survey

A group of patients were recruited from the acute, medium-stay, and rehabilitation wards of the Department of Adult Psychiatry and the Department of Forensic Psychiatry of Castle Peak Hospital (CPH) over a 6-month period as participants to complete the survey questionnaire (Additional file 2). The inclusion criteria were ethnic Chinese aged 18 to 64 years and were able and willing to provide written informed consent to participate in the study. Those with moderate grade mental deficiency and were not able to comprehend Chinese were excluded. All the participants had signed a written informed consent for the participation in this study. Socio-demographic and clinical characteristics of the participants were obtained.

In the survey questionnaire, the participants were asked to choose ten items of information provision and ten items of patient’s participation that were perceived by them as the most important. They were then asked to arrange the items in rank order with one representing the most important and ten representing the least important among the ten important items for information provision and patient’s participation.

### Statistical analysis

Survey data were analysed with the Statistical Package for Social Sciences, Windows version 17.0. Descriptive statistics were applied for data analysis of the questionnaire survey and Chi-square tests with cross tabulation were adopted to explore whether there were any differences across the results of the questionnaire survey in terms of gender, age group, educational level, and diagnosis of the participants. Significant levels were set at *p* < 0.05 throughout the study.

## Results

### Survey on needs of psychiatric inpatients

A convenience sample of 183 inpatients recruited from the acute wards, medium-stay wards, and rehabilitation wards of CPH completed the survey questionnaire on the importance of different items concerning information and participation needs. More than half (60.1 %) of the inpatients were males. The largest group by age (29.0 %) fell into the age range of 31 - 40 years and most (68.3 %) of the total sample had a diagnosis of schizophrenia (Table 3).

**Table 3:** Demographic characteristics of participants in the questionnaire survey

	Number	Percentage
Total	183	100
Gender		
Male	110	60.11
Female	73	39.89
Age		
18–30	39	21.31
31–40	53	28.96
41–50	45	24.59
51–60	41	22.40
> 60	5	2.73
Primary Diagnosis		
Schizophrenia	125	68.31
Depression/Bipolar affective disorder/Schizoaffective disorder	58	31.69
Education Level		
No formal education	5	2.73
Primary	28	15.30
Secondary	141	77.05
Tertiary	9	4.92
Current wards/units		
Acute wards	35	19.13
Medium-stay wards	93	50.82
Rehabilitation wards	55	30.05

### Information needs

For information needs, the top five needs rated by patients as the most important in descending order were: “Information on the classifications of mental illnesses, signs and symptoms and factors contributing to relapse” (frequency of being rated as important: 125), “Information on the criteria and arrangements for discharge” (frequency of being rated as important: 112), “Information on the importance of psychiatric drug taking and its side effects” (frequency of being rated as important: 109), “Information on hospital fees and application of financial assistance” (frequency of being rated as important: 107), and “Information on daytime training placements after discharge from inpatient setting” (frequency of being rated as important: 95) (Table 4).

**Table 4:** Information needs

Information needs (22 items)	Frequency (*n* = 183)	Frequency rank
Information on the classifications of mental illnesses, signs and symptoms and factors contributing to relapse	125	1
Information on the reasons for in-patient admission and related ordinance	93	6
Information on different residential placements	90	10
Information on daytime training placements after discharge from in-patient setting	95	5
Information on the criteria and arrangements for discharge	112	2
Information on the ways to raise self-image and overcome stigma adhered to people with mental illnesses	93	7
Information on hospital fees and application of financial assistance	107	4
Information on the reasons behind the hospital rules and regulations	65	
Information on the daily routine and schedules of ward	76	
Information on how to maintain good physical health	91	9
Information on the arrangements at different stages of rehabilitation including pre-discharge arrangements	82	
Information on the news from the outside world	72	
Information on patient’s right (including ways of expressing opinion and making enquiries)	93	8
Information on how relatives can accept, participate and help	76	
Information on job acquisition	87	
Information on stress management and relapse prevention	81	
Information on alcohol and substance misuse	42	
Information on the schedule of meetings with healthcare professionals	60	
Information on community services and resources	57	
Information on the different kinds of care and rehabilitation services	46	
Information on medication prescriptions	59	
Information on the importance of psychiatric drug taking and its side effects	109	3

There were no significant differences between different genders (male vs female) on the ratings of the items on information needs. Concerning the ratings by different age groups (aged 40 years or below vs aged >40 years), significantly more participants in the younger age group rated “Information on medication prescriptions” as important (χ^2^(1, *N* = 183) = 22.44, *p* = .004). For ratings by different educational groups (primary educational level or less vs secondary educational level or above), significantly more participants with a lower educational level rated “Information on the daily routine and schedules of ward” as important (χ^2^(1, *N* = 183) = 18.88, *p* = .026). For diagnostic groups (schizophrenia vs non-schizophrenia), significantly more participants with a diagnosis of schizophrenia rated “Information on the importance of psychiatric drug taking and its side effects” as important (χ^2^(1, *N* = 183) = 17.32, *p* = .044).

### Participation needs

For participation needs, the top five needs rated by patients as the most important in descending order were: “Enquiring about personal needs and arrangements” (frequency of being rated as important: 142), “Keeping in touch with the outside world” (frequency of being rated as important: 135), “Learning and practising self-management” (frequency of being rated as important: 132), “Collaborating with professionals in setting treatment and care plans” (frequency of being rated as important: 129), and “Reporting discomfort actively” (frequency of being rated as important: 121) (Table 5).

**Table 5:** Participation needs

Participation needs (16 items)	Frequency (*n* = 183)	Frequency rank
Collaborating with professionals in setting treatment and care plans	129	4
Learning and practising self-management	132	3
Enquiring about personal needs and arrangements	142	1
Providing opinion on how to improve the services and facilities of ward	107	
Keeping in touch with the outside world	135	2
Expressing needs and following up whether needs are meet	119	6
Understanding the treatment and rehabilitation plans and providing enough information for healthcare professionals to take reference	95	
Learning from other patients	91	
Reporting discomfort actively	121	5
Expressing personal feelings	118	7
Preparing for the interview with healthcare professionals	90	
Learning how to cope with stress and prevent relapse	106	
Participating in vocational training	116	9
Participating in social activities	118	8
Participating in training of domestic and self-care skill	111	10
Learning how to take care of others	78	

There were no significant differences between different genders (male vs female) and different educational groups (primary educational level or less vs secondary educational level or above) on the ratings of the items on participation needs. Concerning the ratings by different age groups (aged 40 years or below vs aged >40 years), significantly more participants in the younger age group rated “Keeping in touch with the outside world” as important (χ^2^(1, *N* = 183) = 23.85, *p* = .005). For diagnostic groups (schizophrenia vs non-schizophrenia), significantly more participants with a diagnosis of schizophrenia rated “Understanding the treatment and rehabilitation plans and providing enough information for healthcare professionals to take reference” as important (χ^2^(1, *N* = 183) = 24.05, *p* = .004).

## Discussion

### Recovery-oriented mental health service

In the implementation of recovery-oriented mental health service, “patient’s empowerment with the provision of information” and “patient’s participation’ should be the strategies that are promoted by all those involved in care delivery. Exploration of the information needs and participation needs of Chinese psychiatric inpatients should be an important step for the implementation of recovery-oriented mental health service. This qualitative and quantitative study investigated the information needs and participation needs of Chinese psychiatric inpatients.

### Information needs

Results of this study revealed that “Information on the classifications of mental illnesses, signs and symptoms and factors contributing to relapse”, “Information on the criteria and arrangements for discharge”, “Information on the importance of psychiatric drug taking and its side effects”, “Information on hospital fees and application of financial assistance”, and “Information on daytime training placements after discharge from in-patient setting” were rated as the most important information that Chinese psychiatric in-patients perceived that they needed to have during their hospital stay.

Surveys reveal that meeting the information needs and participation needs of patients increases their satisfaction for the services [9, 10, 19, 20]. Moreover, providing psychiatric patients with information about treatment and involving them in care decisions during inpatient care may help to facilitate the transition from inpatient to outpatient settings [30]. On the other hand, inadequacy of information and the consequent exclusion from discussions and decisions about treatment are considered as enduring complaints by service users about mental health services [18].

A recent patient satisfaction survey in Castle Peak Hospital pointed out the needs for further improvement in information provision and participation [31]. The survey also highlighted the concern about health information available to patients on both the quantity of information (e.g., amount of information, number of sources, types and strategies for distribution) and the quality of information (e.g., validity, relevance, accessibility, understandability, timing of acquisition) [31].

The results of the current study were in line with the findings of studies in other countries on non-Chinese populations [32–34]. In a study on the educational needs of chronic psychiatric patients, it was found that patients reported strong interest in learning more about psychiatric illness [32]. In another study on the quality of psychiatric inpatient care, conducted in the United Kingdom, it was found that providing patients with information on health conditions, psychiatric medications, and care after discharge was important in the delivery of high quality mental health care [33]. A survey conducted in Germany on the indicators of quality of inpatient psychiatric treatment from the patients’ view revealed that information about the effects and side effects of medications as well as the effectiveness of medications were rated as a significantly important indicator [34]. The results of the current study also showed that patients with different age groups, educational levels, and diagnoses had different information needs so the provision of information should be based on the different socio-demographic and clinical characteristics of the patients. The current study showed that younger patients were more concerned about information on the medications prescribed to them. Younger patients in this study may be in an earlier stage of mental illness so that they may be more concerned about the information on the medications which they need to be taken for the subsequent months or years. In the current study, patients with lower educational level rated the information on the daily routine and schedule of ward as important to them. This result indicates that patients with lower educational level may be more concerned about basic ward information such as the time for waking up and going to bed as well as time for meals and bathing whereas those with higher educational level may require more information on their mental illness and medications. Also, patients with schizophrenia were more concerned about the side effects of the medications prescribed to them. This result can be explained by the obvious potential side effects of antipsychotics such as extra-pyramidal side effects as compared with that caused by medications for treatment for other diagnoses such as antidepressant for depression.

The results of the present study serve as a reference for the design of information materials in order to meet the information needs of Chinese psychiatric inpatients. As for the delivery of information in psychiatric inpatient setting, it is certainly a task for multi-disciplinary input. For examples, case medical officers provide information on medications; nurses provide information on mental health; occupational therapists provide information on vocational placements; and medical social workers provide information on financial support. Psychiatric nurses have a significant role to take in the empowering process due to their long duration of contact with the patients in day-to-day nursing care at wards, while the other professionals also play very important roles to empower patients in different degrees during patient contacts.

### Participation needs

The participation and involvement of service users in organisational service planning and services’ clinical governance processes has been highlighted in the literature on quality of psychiatric inpatient care [8]. In addition to collaboration in their care, service users’ involvement in developing, running and reviewing services has been shown to have positive outcomes for both the services and the service users who take on these roles [9, 10]. Chinese psychiatric patients are relatively passive and seldom speak up and express their needs. In contrast, traditional Chinese mental health professionals tend to adopt the “paternalistic” or “maternalistic” model of care and design care plans for patients based on the professionals’ own perspective [24]. Patients’ participation in their care in psychiatric inpatient settings is therefore a challenge for both patients and mental health professionals in the Chinese populations.

Results of this study revealed that “Enquiring about personal needs and arrangements”, “Keeping in touch with the outside world”, “Learning and practising self-management”, “Collaborating with professionals in setting treatment and care plans”, and “Reporting discomfort actively” were rated as the most important participation needs for Chinese psychiatric inpatients. These results showed that despite Chinese psychiatric inpatients being relatively passive, they still rated collaboration with healthcare professionals in deriving their treatment and care plan as important. The results of the current study were in line with other studies on non-Chinese populations that psychiatric inpatients do consider that their participation in their treatment plan as important [32, 34–36]. In a study conducted in America on shared decision-making preferences of patients with severe mental illness, most of the patients preferred shared decision making, particularly in relation to their mental health care [35]. Another study conducted in America on the need of chronic psychiatric inpatients revealed that patients had strong interest in learning strategies and skills for coping with common personal problems [32]. A study conducted in Germany showed that patient’s participation in medication decisions was rated as a significant indicator for high quality psychiatric inpatient care [34]. In contrast to other studies on non-Chinese populations in which psychiatric patients wanted a more active role in psychiatric service planning and policy making, the Chinese patients in our present study were concerned much more with their personal needs and self-management, as well as their participation in their own treatment plan [37].

Basing on the results of the current study, the participation needs of Chinese psychiatric inpatient might be met by making posters, pamphlets, videos to encourage patients to speak up and participate, by encouraging patients to join multi-disciplinary clinical case round, and by involving patients in the early stage of care plan compilation. On the other hand, training should be provided to patients on their self-management skills and domestic skills. The results of the current study showed that patients from different age groups and patients with different psychiatric diagnoses had different participation needs and a significant number of patients, especially those with a younger age, rated keeping in touch with the outside world as important. Despite there has been no study on Chinese populations showing similar finding, the finding in this study that younger patients wanted to keep in touch with the outside world can be explained by the rapid developments of information technology such as internet and facebook that patients, especially those with younger age, would like to continue to get access to different information that they could have access to before they are hospitalised. Therefore, inpatient programmes should be tailored made according to the different socio-demographic and clinical characteristics of inpatients such as their age groups and diagnostic groups and should also include community orientation and news reading or internet surging in order to keep the inpatients in touch with the outside world. The current study also revealed that patients with schizophrenia were more concerned about participation in their rehabilitation plan. Despite there has been no similar result found in the Chinese populations in previous studies, this result highlighted the importance of involving schizophrenia patients in the design and revision of their rehabilitation plan.

### Limitations and suggestions for future research

This study had several limitations. The sample size was small and only adult Chinese inpatients in a mental hospital were recruited with the exclusion of child and adolescent patients and elderly patients. Generalisability of the study is therefore limited. Patients rated the importance of their information needs and participation needs basing on a questionnaire with a fixed list of items identified by focus group discussion. It might have been worthwhile to allow patients to write in the questionnaire in an open-ended format if they think that there are any other areas of information needs and participation needs that are important but are not addressed in the list in the questionnaire. A future study with a larger sample size is warranted in order to analyse whether Chinese inpatients from acute wards, medium-stay wards, and rehabilitation wards have different information and participation needs. This was a cross-sectional study with the inpatients rated their information and participation needs at one particular time point of their illness stage. A longitudinal study could be conducted to follow up the patients throughout their pathway of inpatient care to explore how their information and participation needs change as they go through different stages of their mental illness (e.g., at the acute, subacute, and chronic stages of illness) and its treatment. Moreover, the psychometric properties of the survey questionnaire were not thoroughly tested in the present study. It would be worthwhile to recruit a larger sample in order to perform factor analysis or Rasch analysis as well as test-retest reliability analysis for the exploration of the validity and reliability of the survey questionnaire in future. A longitudinal study is also warranted to assess the sensitivity to changes of the survey questionnaire.

## Conclusions

The concept and construct of recovery, despite being rather abstract, has been evident in patient’s self-help since the 1930s and gained its prominence in the mental health literature since the 1980s [38]. The adoption of the concept of recovery in mental health service transformation and delivery has been widely discussed in the literature in the recent years [39–41]. The incorporation of the recovery principles into the government reports and policy statements on mental health service in many countries as well as the rapid emergence of studies on mental health recovery point to the urgent need to explore culturally-relevant ways of implementing mental health recovery-oriented practice within Chinese populations [1–7, 11, 42–46]. Apart from being in line with the global trend of mental health service reform, the implementation of recovery-oriented practices also matches well with the increasing emphasis of human rights and patient’s involvements in the Chinese society [24]. Meeting the information needs and participation needs of psychiatric inpatients are salient for the delivery of quality psychiatric inpatient care and is proven to improve patients’ outcomes. This is the first study to explore the information needs and participation needs of Chinese psychiatric inpatients in this locality. This study reveals that Chinese psychiatric inpatients are concerned about information on their mental illness and its treatments as well as the criteria for discharge. On the other hand, patients are concerned about their personal needs, their self-management, as well as their keeping in touch with the outside world during their hospitalisation. Moreover, patients with different socio-demographic and clinical characteristics have different information and participation needs. The results of the present study serve as a reference for mental health professionals and policy makers to design strategies, guidelines, service models, and programmes in order to fulfil the information needs and participation needs of Chinese psychiatric inpatients in Hong Kong.

## Additional files

Additional file 1: Focus Group Discussion Guide. This guide included a list of guiding questions and a list of non-directive probes to guide the focus group discussion. (DOCX 18 kb) Additional file 2: Survey Questionnaire on Information and Participation needs. This survey questionnaire was developed basing on the item pool obtained on information provision and patient’s participation in the focus group discussion and was the assessment tool adopted in the second part of the study. (DOCX 19 kb)
