# Supplementary material for: Pathway to mental health recovery: a qualitative and quantitative study on the needs of Chinese psychiatric inpatients
Source: BMC Psychiatry. 2016 Jul 12;16:236. doi: 10.1186/s12888-016-0959-6 (PMC4942969; doi:10.1186/s12888-016-0959-6)
Supplement: Additional file 1: — Focus Group Discussion Guide. This guide included a list of guiding questions and a list of non-directive probes to guide the focus group discussion. (DOCX 18 kb) [file 12888_2016_959_MOESM1_ESM.docx]

**Additional file 1**

**Focus Group Discussion Guide**

1. Each focus group discussion will be conducted with one moderator (investigator) and one co-moderator (research assistant).
2. Focus group members and moderators will be arranged to seat in a circle to facilitate interaction.
3. The focus group discussion will be conducted in Cantonese. The procedure of running the focus group discussion will be divided into 3 parts.

**Part 1 – Introduction and Warn up (20 minutes)**

1. The moderators should introduce themselves to the group members and welcome everyone to the group.
2. The moderators should then explain the purpose and the logistics of the focus group discussion. Written informed consents will then be obtained from each focus group member.
3. The moderators should emphasise that group members are free to share their views and that their views would be audio-recorded, and in the recordings, none of the focus group members will be identified individually.
4. Audio-recording should then be started to facilitate data collection and all the focus group members will be clearly informed of the recording.
5. A simple warm-up activity should then be introduced. Each focus group member should be encouraged to share briefly about their experience of past and current in-patient admissions.

**Part 2 – Discussion on needs (60 minutes)**

1. After the warm-up activity, group members should be guided to share their views about the needs for information and participation during their course of in-patient treatment with non-directive probes.

**Part 3 – Round up (10 minutes)**

1. Moderators should thank for the focus group members’ participation.
2. Moderators should briefly summarise some key points discussed.
3. Focus group members should be briefed again on the purpose of the focus group discussion and be provided with the opportunities for their final questions before dismissing.

**List of guiding questions:**

1. What do you think about the information needs of patients during their hospital admission period?
2. What do you think about the participation needs of patients in different aspects during their hospital admission period?
3. What information do you expect to know during your (patient’s) in-patient treatment?
4. What types/opportunities of participation do you expect to have during your (patient’s) in-patient treatment?
5. Do you think the information provided to you is adequate during your (patient’s) hospitalisation? If not, how to improve?
6. Do you think the types of/opportunities for your participation are adequate during your (patient’s) hospitalisation?
7. Can you think of some good/bad experiences about getting information during your (patient’s) hospitalisation?
8. Can you think of some good/bad experiences about personal participation during your (patient’s) hospitalisation?
9. What is the information you think to be important to you (patient) during hospitalisation?
10. What are the types of/opportunities for participation you think to be important to you (patient) during hospitalisation?

**List of non-directive probes:**

1. Can you tell me why?
2. Can you elaborate or explain in more details?
3. Any additional comments or opinions?
4. How about the other members’ view?
