# Supplementary material for: Pathway to mental health recovery: a qualitative and quantitative study on the needs of Chinese psychiatric inpatients
Source: BMC Psychiatry. 2016 Jul 12;16:236. doi: 10.1186/s12888-016-0959-6 (PMC4942969; doi:10.1186/s12888-016-0959-6)
Supplement: Additional file 2: — Survey Questionnaire on Information and Participation needs. This survey questionnaire was developed basing on the item pool obtained on information provision and patient’s participation in the focus group discussion and was the assessment tool adopted in the second part of the study. (DOCX 19 kb) [file 12888_2016_959_MOESM2_ESM.docx]

**Additional file 2**

**Survey Questionnaire on Information and Participation needs**

**Patient’s name：_________________ Ward：_____________ Date：＿＿＿＿＿**

**Information needs**

Instructions:

Step 1： Among the 22 items of information needs of Chinese psychiatric in-patients, please select the 10 items that are perceived by you as the most important by putting a ✓ in the column

Step 2： For the 10 selected items in step 1, please arrange the items in rank order with 1 representing the most important and 10 representing the least important among the 10 important items

| Information needs (22 items) | Step 1: Please select 10 most important items by putting a ✓ | Step 2: Please rank the 10 selected items in step 1 in rank order |
| --- | --- | --- |
| Information on the classifications of mental illnesses, signs and symptoms and factors contributing to relapse |  |  |
| Information on the reasons for in-patient admission and related ordinance |  |  |
| Information on different residential placements |  |  |
| Information on daytime training placements after discharge from in-patient setting |  |  |
| Information on the criteria and arrangements for discharge |  |  |
| Information on the ways to raise self-image and overcome stigma adhered to people with mental illnesses |  |  |
| Information on hospital fees and application of financial assistance |  |  |
| Information on the reasons behind the hospital rules and regulations |  |  |
| Information on the daily routine and schedules of ward |  |  |
| Information on how to maintain good physical health |  |  |
| Information on the arrangements at different stages of rehabilitation including pre-discharge arrangements |  |  |
| Information on the news from the outside world |  |  |
| Information on patient’s right (including ways of expressing opinion and making enquiries) |  |  |
| Information on how relatives can accept, participate and help |  |  |
| Information on job acquisition |  |  |
| Information on stress management and relapse prevention |  |  |
| Information on alcohol and substance misuse |  |  |
| Information on the schedule of meetings with healthcare professionals |  |  |
| Information on community services and resources |  |  |
| Information on the different kinds of care and rehabilitation services |  |  |
| Information on medication prescriptions |  |  |
| Information on the importance of psychiatric drug taking and its side effects |  |  |

**Participation needs**

Instructions:

Step 1： Among the 16 items of participation needs of Chinese psychiatric in-patients, please select the 10 items that are perceived by you as the most important by putting a ✓ in the column

Step 2： For the 10 selected items in step 1, please arrange the items in rank order with 1 representing the most important and 10 representing the least important among the 10 important items

| Participation needs (16 items) | Step 1: Please select 10 most important items by putting a ✓ | Step 2: Please rank the 10 selected items in step 1 in rank order |
| --- | --- | --- |
| Collaborating with professionals in setting treatment and care plans |  |  |
| Learning and practising self-management |  |  |
| Enquiring about personal needs and arrangements |  |  |
| Providing opinion on how to improve the services and facilities of ward |  |  |
| Keeping in touch with the outside world |  |  |
| Expressing needs and following up whether needs are meet |  |  |
| Understanding the treatment and rehabilitation plans and providing enough information for healthcare professionals to take reference |  |  |
| Learning from other patients |  |  |
| Reporting discomfort actively |  |  |
| Expressing personal feelings |  |  |
| Preparing for the interview with healthcare professionals |  |  |
| Learning how to cope with stress and prevent relapse |  |  |
| Participating in vocational training |  |  |
| Participating in social activities |  |  |
| Participating in training of domestic and self-care skill |  |  |
| Learning how to take care of others |  |  |

For staff use only

Patient Name (in English) ____________________________

(in Chinese) __________________________

Ward: ___________ Age / Sex: ________________

Hospital number: _________________________________

Primary Diagnosis:　🞏 Schizophrenia 🞏 Other, please specify: ________________________________

Education Level: 🞏 No formal education 🞏 Primary 🞏 Secondary 🞏 Tertiary

Date of admission: _________________ 1^st^ admission to CPH? 🞏 Yes 🞏 No

Questionnaire completed on _________________ (Date)

as assisted by ___________________________________ (Name of staff)
